# In Silico Identification of the NLRP3 Inhibitors from Traditional Chinese Medicine

**DOI:** 10.3390/ijms262110569

**Published:** 2025-10-30

**Authors:** Shunjiang Jia, Huanling Lai, Xinyu Chen, Jiajie Lu, Wei Ding, Dongxiao Cui, Peng Zhao, Qiao Zhang, Yuwei Wang, Chunsong Cheng

**Affiliations:** 1 College of Pharmacy, Shaanxi University of Chinese Medicine, Shiji Ave, Xi’an-Xianyang New Economic Zone, Xianyang 712046, China; 2 Guangzhou National Laboratory, Guangzhou 510000, China; 3 State Key Laboratory of Quality Research in Chinese Medicine, Macau University of Science and Technology, Macau 999078, China; 4 Jiangxi Key Laboratory for Sustainable Utilization of Chinese Materia Medica Resources, Lushan Botanical Garden, Chinese Academy of Sciences, Jiujiang 332900, China

**Keywords:** NLRP3, TCM database, virtual screening, molecular dynamics simulation

## Abstract

NOD-like receptor protein 3 (NLRP3) inflammasome is a key mediator of inflammation and a promising therapeutic target. However, the discovery of novel and effective inhibitors of NLRP3 remains limited. A combined docking-based virtual screening (DBVS) and shape-based screening approach was applied to eight traditional Chinese medicine (TCM) databases to identify potential NLRP3 inhibitors. Structural similarity analysis, ADMET prediction, and molecular dynamics (MD) simulations were performed to evaluate structural novelty, pharmacokinetic properties, and binding stability. A total of 25 potential NLRP3 inhibitors were identified, each exhibiting docking scores higher than those of the reference inhibitor XE3. Structural similarity analysis revealed that the screened compounds exhibited low similarity to previously reported NLRP3 inhibitors, demonstrating their structural novelty. ADMET evaluation indicated that compounds C2, C3, and C4 exhibited favorable physicochemical and pharmacokinetic properties. Molecular dynamics (MD) simulations demonstrated that the complexes of compounds C2, C3, and C4 with NLRP3 remained stable throughout the simulations, exhibiting limited backbone fluctuations and compact conformations, as indicated by Rg values of approximately 6 Å. Solvent-accessible surface area (SASA) and polar surface area (PSA) analyses suggested that compounds C3 and C4 were tightly solvated and maintained favorable membrane permeability. Notably, binding free energy calculations revealed that all three compounds exhibited stronger binding than XE3, with compound C3 showing the most favorable energy (–48.81 ± 3.89 kcal/mol), indicating a highly stable and energetically preferred interaction with NLRP3. This study identified promising TCM-derived compounds as potential NLRP3 inhibitors, offering new directions for anti-inflammatory drug development.

## 1. Introduction

NOD-like receptor protein 3 (NLRP3) is a pivotal regulatory protein within the inflammasome and serves as a critical modulator in the innate immune response [1]. NLRP3 can recognize a wide range of pathogenic microorganisms and stress-related endogenous signaling molecules, making it an indispensable target for maintaining internal homeostasis [2]. Existing studies have demonstrated that dysregulated NLRP3 inflammasome activation is closely associated with the pathogenesis of various major human diseases, including atherosclerosis, hypertension, type 2 diabetes, Alzheimer’s disease, and gout [3,4]. Consequently, the development of inhibitors targeting the NLRP3 inflammasome has emerged as a prominent research focus in both academia and industry [5]. However, due to its intricate structure, the NLRP3 inflammasome has been considered a challenging therapeutic target, with no approved inhibitors currently available [6,7,8].

Chronic diseases have emerged as a significant global health challenge [6]. Malfunction of NLRP3 may contribute to the pathogenesis of various chronic diseases, including chronic inflammatory conditions associated with lifestyle and dietary factors, aging, and environmental exposures, such as diabetes, Alzheimer’s disease, systemic lupus erythematosus, gout, non-alcoholic steatohepatitis (NASH), inflammatory bowel disease, and atherosclerosis. Consequently, modulation of inflammasome activation is crucial for maintaining immune homeostasis and ensuring proper immune function [9].

**Figure 1:**
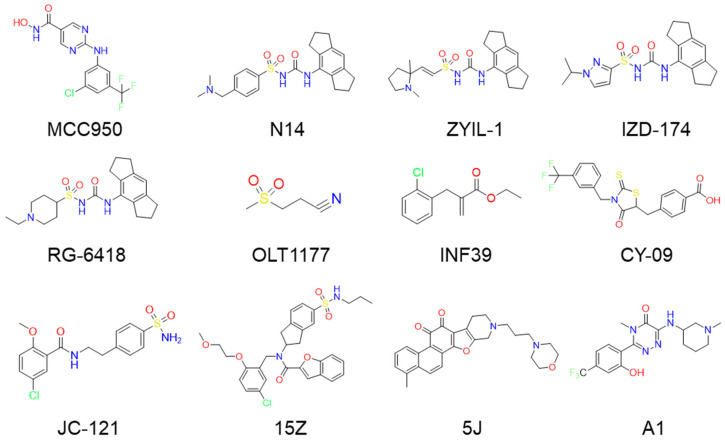
The representative NLRP3 inhibitors.

So far, 12 compounds have entered clinical research. As shown in Figure 1, Olatec reported that Dapansutrile has entered Phase II clinical trials for acute gout, indicating that it can reduce joint pain by more than 50% after three days of treatment [10]. NodThera recently announced positive results from a Phase 1b/2a trial of its NLRP3 inflammasome inhibitor NT-0796 in patients with Parkinson’s disease (PD). The analysis showed that NT-0796 effectively reduced key inflammatory and neuroinflammatory biomarkers in both blood and cerebrospinal fluid (CSF) [11]. Additionally, RRx-001, ZYIL1, HT-6184 and other NLRP3 inhibitors are in the clinical research stage [12]. MCC950/CRID3 is a typical diaryl sulfonylurea inhibitor targeting the NACHT domain of NLRP3, which directly targets the ATP hydrolytic domain of NLRP3 and renders it inactive [13]. Tranilast, a tryptophan metabolite analogue, modulates NLRP3 oligomerization independently of its ATPase activity and suppresses inflammasome activation [14]. β-Hydroxybutyrate, a metabolic product, has been reported to inhibit NLRP3 inflammasome activation and prevent LPS-induced memory impairment [15]. As a potent NLRP3 inhibitor, UK5099 can effectively inhibit the production of IL-1β mediated by NLRP3 inflammasome both in vivo and in vitro, which providing a candidate for the clinical treatment of NLRP3 inflammasome-associated inflammatory diseases [16]. Sorbremnoid A exerts a potent effect of inhibiting the assembly and activation of the NLRP3 inflammasome by directly targeting the NLRP3 protein, which showing a favorable effect of promoting wound healing in the refractory wound healing model of diabetic mice [17]. Nowadays, some traditional Chinese medicine (TCM) components and natural products have been reported to regulate the activation of NLRP3 and thereby interfere with the activation of inflammasome. Saponin Rg3 extracted from ginseng, Oridonin, Artemisinin, Britannin and other natural products [18]. Although a number of inhibitors targeting NLRP3 inflammasome have entered clinical studies, not one small-molecule drug has been approved by FDA for marketing. At the same time, most of the NLRP3 inhibitors entering clinical research are MCC950 analogues, which have the issues of single skeleton and severe structural homogeneity. Therefore, it is of great significance to search for novel and efficient NLRP3 inhibitors.

In the present study, 25 NLRP3 inhibitors were identified from a virtual library constructed from eight commonly used TCM databases by integrating Docking-based virtual screening (DBVS) and shape-based screening. Subsequently, ADMET predictions were performed for the top eight compounds exhibiting the highest shape similarity. To further investigate the binding modes and stability of C2, C3, and C4 within the NLRP3 protein, 200 ns molecular dynamics (MD) simulations were conducted. These findings provide new insights into the design of NLRP3 inhibitors and will facilitate their rational design and pharmacological evaluation for the treatment of human diseases.

## 2. Results

### 2.1. Glide Docking and Crystal Selection

**Figure 2:**
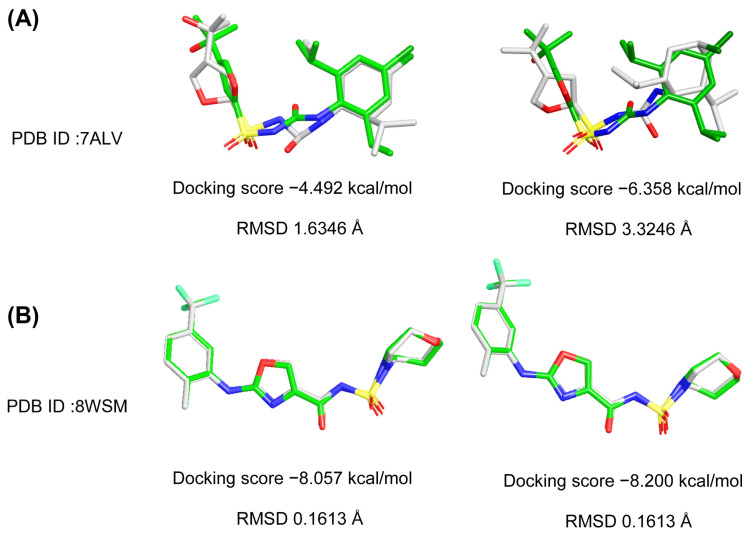
Superimposition of the original ligands with their docked poses. (**A**) SP docking and XP docking of ligand RM5 in NLRP3 protein (PDB ID: 7ALV). (**B**) SP docking and XP docking of ligand XE3 in NLRP3 protein (PDB ID: 8WSM).

To validate the reliability of the docking protocol, Schrödinger 2015 was employed to redock the co-crystallized ligands of NLRP3 crystal structures (PDB IDs: 8WSM and 7ALV). The root-mean-square deviation (RMSD) between the docked and original ligand conformations was calculated to assess docking accuracy. As shown in Figure 2, 8WSM exhibited lower docking scores of −8.057 kcal/mol and −8.200 kcal/mol compared with 7ALV in standard precision (SP) and extra precision (XP) modes, respectively. Furthermore, 8WSM showed lower RMSD values of 0.1613 Å in both SP and XP modes compared with 7ALV. Based on both docking scores and RMSD values, the superimposed RMSD plot is presented in Figure 2. The superimposed structures demonstrated that the original ligand and the docked pose in 8WSM were almost perfectly overlapped. Therefore, the NLRP3 protein (PDB ID: 8WSM) was ultimately selected as the target for virtual screening (VS).

### 2.2. Results of Docking-Based Virtual Screening

A total of 146,730 molecules obtained from the virtual library were screened against the 8WSM structure using various precision modes described in the methodology. As a result, 43,678 ligands were obtained after HTVS for subsequent screening, and 8724 candidates were obtained through SP docking. Finally, 1746 candidates targeting NLRP3 were identified using XP docking. In addition, the docking score of XE3 obtained from XP docking in 8WSM (−8.200 kcal/mol) was used as the screening criterion for further screening. Finally, 1509 compounds with docking scores higher than that of XE3 were retained as candidate compounds.

### 2.3. Results of Shape-Based Screening

Shape-based screening identifies potential molecules by evaluating the degree of shape complementarity between the ligand and the target binding site [19]. Molecules from the virtual library were rapidly screened using the shape-based method, thereby reducing the time and cost required for experimental validation [20]. This approach is particularly advantageous in the early stages of drug screening, as it can greatly enhance screening efficiency. Table 1 summarizes the PubChem ID, compound name, molecular weight (MW), chemical structure, docking score, shape similarity (shape sim), and database source of the top eight compounds. The shape similarity values of C1, C2, C3, and C4 were the highest, with scores of 0.328, 0.305, 0.280, and 0.251, respectively. Among them, C1 (L-malic acid 2-O-gallate) was obtained from the iTCM database and is derived from the active ingredient of *Phyllanthus emblica* L. C2, identified as Dioncophyllinol B, was retrieved from the HERB database and represents the active constituent of trileaf wood. C3, identified as Isoamericanol A, was obtained from the HERB database and originates from an active constituent of *Phytolacca americana* L. C4 was also retrieved from the HERB database; however, its specific natural source has not been clearly documented.

### 2.4. The Calculation of Molecular Similarity

**Figure 3:**
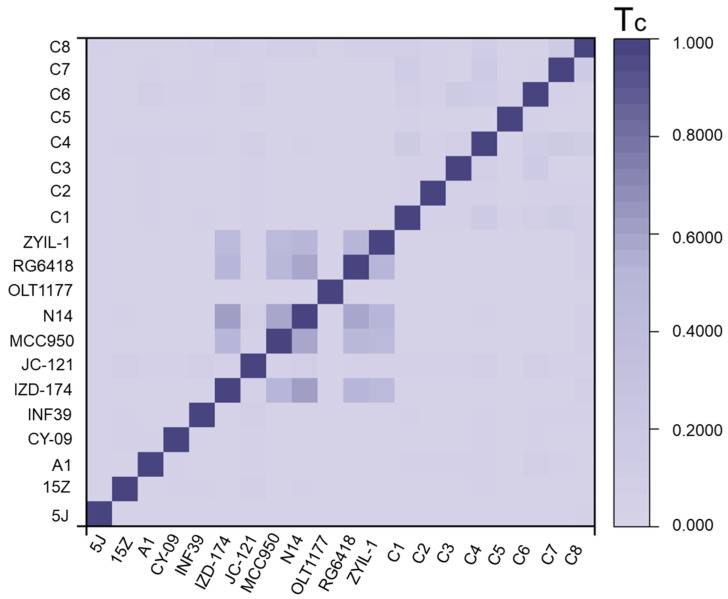
The heatmap of Tc between the existing NLRP3 inhibitors and the screening of NLRP3 inhibitors. In the plot, darker colors indicate regions of higher similarity.

To evaluate the structural novelty of the selected small molecules, their similarity to previously reported NLRP3 inhibitors was calculated. As shown in Figure 3, the structural similarity index (Tc) between the top eight compounds obtained through VS and the NLRP3 inhibitors reported in previous studies was relatively low, indicating that the newly identified inhibitors possessed distinct structural frameworks compared with those previously discovered through experimental approaches.

### 2.5. The Analysis of Pharmacokinetic Properties

**Figure 4:**
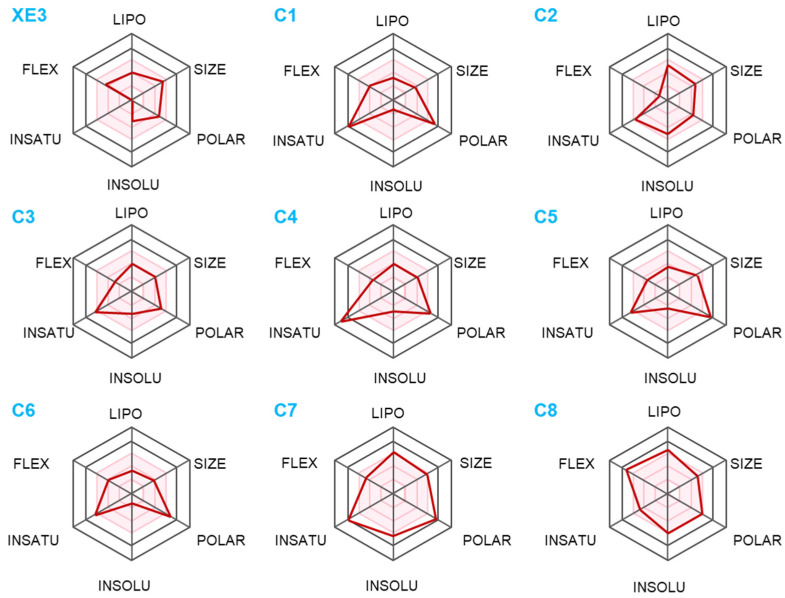
The pharmacokinetic of ligands obtained from SwissADME. The pink area indicates the optimal range for each property.

Predicting and optimizing ADMET characteristics can improve the success rate of drug development [21]. Understanding and optimizing the insolubility of candidate drugs are critical for enhancing their clinical efficacy and successful development [22]. Figure 4 presents the predicted ADMET profiles of the top eight screened compounds and the positive control XE3. C8 exhibited the highest lipophilicity, which generally indicates better cell membrane permeability and broader tissue distribution; however, excessive lipophilicity may lead to poor solubility, slow metabolism, and potential toxicity. C1 and C6 were slightly less lipophilic, suggesting that these molecules may exhibit higher aqueous solubility. C1, C5, C6, and C7 were more polar and exhibited higher water solubility. Flexibility (FLEX) refers to the ability of a molecule to rotate, bend, or deform within three-dimensional space. The high FLEX value of C8 indicates that the molecule possesses a large conformational space, whereas the remaining compounds fall within the pink region, displaying fewer degrees of freedom and relatively rigid structures.

The ADMET parameters calculated by QikProp are summarized in Table 2. QPlogPo/w represents the logarithm of the partition coefficient between octanol and water, which reflects the balance between the lipophilicity and hydrophilicity of a compound [23]. C2 and C7 exhibited higher lipophilicity, facilitating their diffusion across the phospholipid bilayer and potentially enhancing their pharmacological activity. In contrast, C1 displayed better hydrophilicity. QPlogS was used to predict the aqueous solubility of the compounds [24]. and the solubility values of C1–C8 were all within the acceptable range defined by the software. PlogHERG is an important index for evaluating the cardiac safety of drug candidates. PPCaco denotes the permeability of compounds through Caco-2 cell monolayers, which is commonly used to assess intestinal absorption capacity [25]. Accordingly, C2 and C3 showed higher PPCaco values, suggesting a stronger ability to penetrate the intestinal barrier. PlogBB measures the potential of compounds to penetrate the blood–brain barrier (BBB) and enter the central nervous system. C1, C5, C6, and C7 exhibited relatively low PlogBB values, implying limited BBB permeability. Overall, among the screened compounds, C2, C3, and C4 exhibited superior pharmacokinetic properties. Their chemical nomenclature are as follows: compound C2 is 4,8-dihydroxy-7-(1-hydroxy-8-methoxy-6-methylnaphthalen-2-yl)-1,3-dimethyl-1,2,3,4-tetrahydroisoquinolin-2-ium; compound C3 is (E)-4-(3-(hydroxymethyl)-7-(3-hydroxyprop-1-en-1-yl)-2,3-dihydrobenzo[b][1,4]dioxin-2-yl)benzene-1,2-diol; and compound C4 is 2-(2-((3,4-dihydroxybenzoyl)oxy)-4,6-dihydroxyphenyl)acetic acid.

### 2.6. The Analysis of RMSD, RMSF, Rg, SASA and PSA

**Figure 5:**
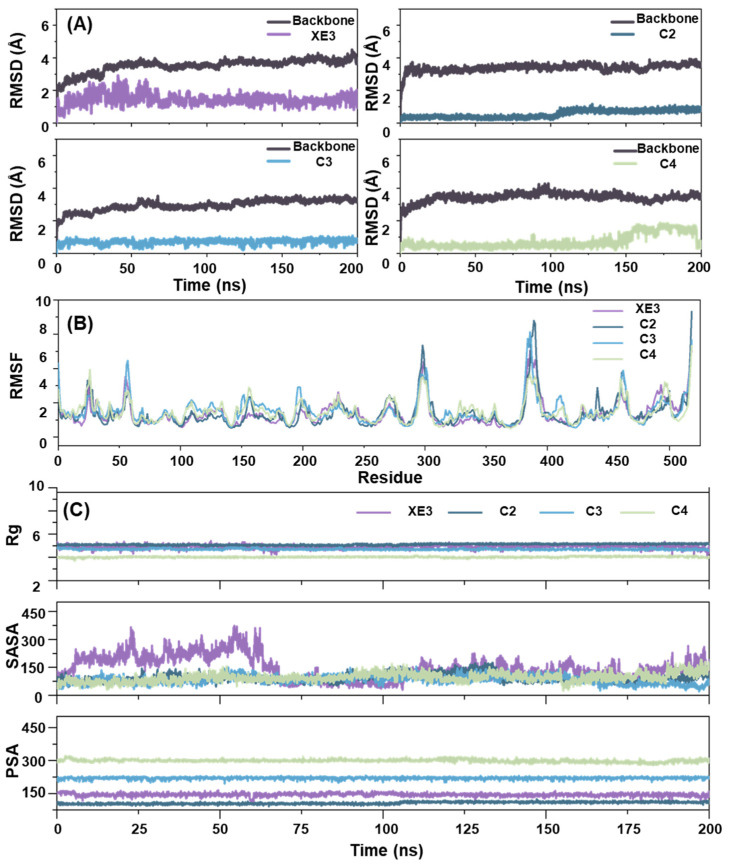
Stability and Properties of ligand-protein complexes determined by MD simulation for 200 ns, including (**A**) RMSD, (**B**) RMSF. (**C**) Rg, SASA, PSA.

In MD simulations, RMSD serves as a quantitative measure to evaluate structural deviations of molecules over time. It is particularly useful for assessing the conformational stability of molecules and their resemblance to reference structures throughout the simulation. By monitoring RMSD values during the simulation, researchers can determine whether the system has reached an equilibrium state [22]. Considering the unfavorable pharmacokinetic properties of C1, MD simulations were conducted for the positive control XE3 and the top three candidates (C2, C3, and C4, based on shape sim) for 200 ns to investigate the stable state of the ligands within the NLRP3 system. As shown in Figure 5A, all complexes exhibited convergence and stabilization during the final 50 ns, confirming the reliability of the MD simulations. Notably, C2 displayed greater stability at the initial stage of the simulation compared with the other complexes. Furthermore, analysis of the backbone fluctuations revealed that the screened compounds exhibited higher structural stability than the positive control XE3 in the NLRP3 system.

Root Mean Square Fluctuation (RMSF) is a quantitative parameter used to evaluate the flexibility of specific atoms, typically focusing on the alpha carbons in MD simulations. This analysis helps identify flexible and rigid regions within the molecule. For proteins, higher RMSF values in particular residues indicate greater flexibility, usually corresponding to loop structures or surface-exposed regions. Conversely, lower RMSF values suggest increased rigidity, which is often associated with secondary structural elements such as alpha-helices and beta-sheets [26]. As shown in Figure 5B, the most pronounced fluctuations were observed in the positive control XE3. Specifically, XE3 exhibited notable fluctuations of 6.876 Å at ASN545 and 6.104 Å at VAL546. In contrast, CYS470 displayed minimal fluctuation with an amplitude of 0.571 Å. The residues exhibiting the largest fluctuations were mainly located in the loop regions of the protein. Moreover, the residues in the C4 system showed smaller fluctuations compared with those in other systems, indicating that the overall complex exhibited lower flexibility and tighter ligand binding during the MD simulation.

Radius of Gyration (Rg) reflected the degree to which a molecule or complex expanded or contracted in three-dimensional space. A smaller Rg value indicated a more compact molecular conformation, whereas a larger Rg value suggested a looser or more extended structure [27]. As shown in Figure 5C, the Rg values of C2, C3, and C4 remained around 6 Å at 200 ns. The Rg value of C4 remained consistently lower, indicating that C4 was more tightly associated with the protein backbone in the NLRP3 system. SASA was an important parameter used to quantify the solvent-accessible surface area of a molecule, particularly for proteins [28]. During the first 20 ns of the MD simulation, the Solvent Accessible Surface Area (SASA) values of all compounds exhibited an increasing trend. However, XE3 displayed an increase in SASA between 15 and 90 ns, which may have resulted from a slight displacement of the water box during this period. This displacement caused certain protein residues to become more solvent-exposed, resulting in higher SASA values. Polar Surface Area (PSA) provided insights into the interaction of molecules with polar environments, such as aqueous solvents. Molecules with larger PSA values were more likely to interact with water or other polar molecules, whereas those with smaller PSA values tended to associate with hydrophobic environments. The XE3–NLRP3 complex exhibited a larger PSA, suggesting that the system was more likely to remain solvated within the water box. Conversely, C2 and XE3 displayed smaller PSA values, suggesting a greater potential to cross cell membranes.

### 2.7. The Analysis of Free Energy Landscape and Binding Mode

**Figure 6:**
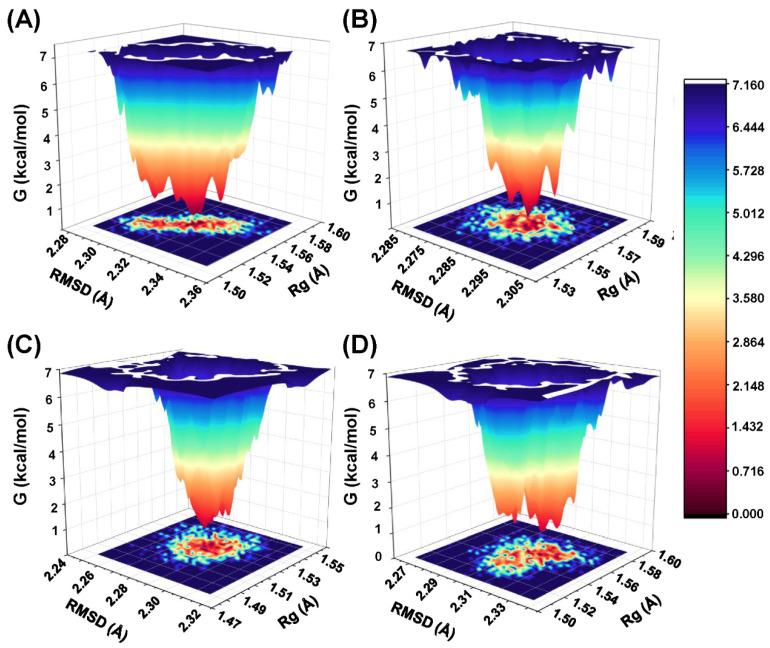
The free energy landscapes of ligands during MD simulations. (**A**) XE3, (**B**) C2, (**C**) C3, and (**D**) C4.

To analyze the energetic basis of protein conformational changes, free energy landscape (FEL) maps were constructed, with each distinct energy minimum representing a unique low-energy conformation. The FEL illustrating the global minima of the protein backbone atoms with respect to RMSD and Rg is presented in Figure 6. In the FEL analysis, the NLRP3 complexes reached their lowest-energy conformations. The conformational transitions within each complex are represented by distinct subspaces, suggesting that these small-molecule inhibitors interact with the protein through diverse binding modes, which may contribute to subtle differences in binding stability.

**Figure 7:**
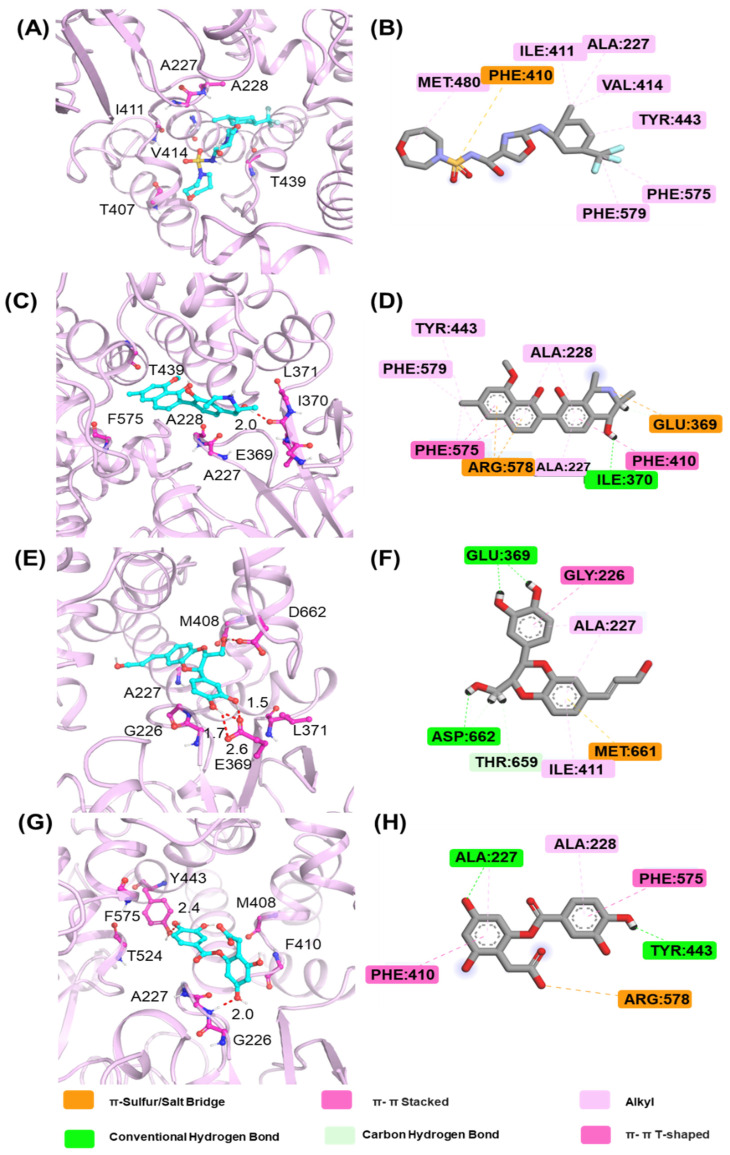
The binding mode of NLRP3 and compounds. The 3D binding mode of (**A**) XE3, (**C**) C2, (**E**) C3, (**G**)C4. The 2D binding mode of (**B**) XE3, (**D**) C2, (**F**) C3, (**H**) C4.

The lowest-energy conformations obtained from the FEL analysis were extracted to investigate the binding modes. The representative conformations of XE3, C2, C3, and C4 are shown in Figure 7. For the positive control XE3, PHE410 forms a π–sulfur interaction with the nitrogen atom, while MET 480, ILE 411, and ALA 277 play key roles in stabilizing the complex. For C2, the six-membered ring containing a nitrogen atom forms a conventional hydrogen bond with ILE 370, and TYR443, PHE579, and ALA 228 exhibit alkyl interactions with ALA227. For C3, the two hydroxyl groups on the catechol moiety form conventional hydrogen bonds with GLU 369, and the hydroxyl group of the six-membered ring containing two oxygen atoms forms a hydrogen bond with ASP 662. Moreover, GLY226, THR 659, and MET 661 also contribute to stabilizing the binding conformation. The phenolic hydroxyl group of C4 forms hydrogen bonds with TYR 443 and ALA 227. During the binding of C4 to the NLRP3 protein, PHE 575, ALA 228, and ALA 227 play key roles in maintaining complex stability.

### 2.8. The Calculation Binding Free Energy

The binding free energies of the ligand–protein complexes were estimated using the MMGBSA method based on the equilibrated MD trajectories, and the results are summarized in Table 3. Among the studied compounds, C3 exhibited the most favorable total binding free energy of −48.81 ± 3.89 kcal/mol, which was lower than that of the positive control XE3 with −42.31 ± 5.31 kcal/mol. This result indicates that C3 forms a more stable and energetically favorable complex with the target protein.

Compound C3 showed the most favorable total binding free energy of −48.81 ± 3.89 kcal/mol, which was lower than that of the reference compound XE3 at −42.31 ± 5.31 kcal/mol. This result indicates that C3 forms the most stable and energetically favorable complex with the target protein. The relatively small standard deviation demonstrates good convergence and stable interactions during the simulation. Energy decomposition revealed that van der Waals interactions contributed −34.60 ± 2.77 kcal/mol and lipophilic interactions contributed −19.61 ± 1.76 kcal/mol, indicating that hydrophobic packing and shape complementarity are the main driving forces for binding. C3 also exhibited the most favorable hydrogen-bonding contribution of −3.04 ± 0.56 kcal/mol, suggesting the presence of stable and directional hydrogen bonds that help anchor the ligand within the binding pocket. The balanced combination of hydrophobic and polar interactions allows C3 to minimize desolvation penalties while maximizing favorable contacts, which explains its superior binding affinity and overall stability. These features make C3 the most promising candidate for further structural optimization.

Compound C2 displayed a total binding free energy of −34.17 ± 5.79 kcal/mol, which was less favorable than that of C3 despite its strong Coulombic interaction of −81.64 ± 10.54 kcal/mol. This large electrostatic contribution suggests strong charge–charge or dipole–dipole interactions between the ligand and the protein. However, in MMGBSA calculations, such strong Coulombic attraction is often counterbalanced by an unfavorable polar solvation effect that arises from the desolvation of charged or highly polar groups.

Although van der Waals and lipophilic interactions contributed −41.06 ± 2.79 and −19.64 ± 1.23 kcal/mol, respectively, these effects could not compensate for the energy loss due to solvation. As a result, the overall binding affinity of C2 remained moderate.

Compound C4 showed the least favorable total binding free energy of −33.50 ± 5.25 kcal/mol, indicating weak stabilization in the active site. The van der Waals and lipophilic contributions, −29.05 ± 2.77 and −13.45 ± 1.75 kcal/mol, suggest limited hydrophobic packing. The Coulombic energy of −3.64 ± 17.94 kcal/mol exhibited large fluctuations, reflecting unstable electrostatic interactions, while the hydrogen-bonding term of −2.21 ± 0.87 kcal/mol indicates only a few weak hydrogen bonds. These results suggest that C4 binds less effectively due to poor geometric complementarity or high conformational flexibility. Enhancing hydrophobic contacts and stabilizing polar interactions could improve its affinity.

Overall, the MMGBSA analysis indicates that C3 achieves the best energetic balance through strong van der Waals and hydrogen-bonding interactions with minimal desolvation effects. C2 shows strong electrostatic attraction but is penalized by solvation, whereas C4 lacks sufficient stabilizing interactions. These findings emphasize the need for a proper balance between hydrophobic and polar forces to achieve effective ligand binding and guide future optimization efforts.

## 3. Discussion

VS is a computational method used to identify compounds with potential biological activity by simulating and predicting their interactions with target biomolecules such as proteins, nucleic acids, enzymes, and receptors. This approach markedly improves the efficiency and success rate of drug discovery and design. In the present study, DBVS combined with shape-based virtual screening was used to identify small-molecule inhibitors targeting NLRP3 from eight Chinese medicine databases. Finally, 25 candidate compounds were obtained for further studies. Notably, the docking scores of all compounds were superior to that of the positive control XE3, providing a solid foundation for subsequent systematic evaluation and prediction of their chemical properties. Similar approaches have been successfully applied in identifying NLRP3 inhibitors such as MCC950 and CY-09, supporting the validity of our computational pipeline [29].

To evaluate the structural novelty of the screened compounds, we calculated the Tc relative to previously reported NLRP3 inhibitors, including MCC950, Oridonin, and CY-09 [30]. The screened compounds exhibited markedly lower Tc values, indicating that they possess distinct chemical scaffolds rather than being simple analogs of known inhibitors. In contrast, many previously reported NLRP3 inhibitors share moderate-to-high structural similarity, which may limit the diversity of their binding modes and therapeutic applications. The low similarity observed in our candidates suggests the potential for novel interactions with NLRP3, possibly enabling unique mechanisms of inhibition and reducing the risk of cross-resistance associated with existing compounds. Furthermore, several natural product-derived NLRP3 inhibitors are valued for their favorable safety profiles, and our structurally novel compounds may similarly provide a balance between efficacy and low toxicity. Overall, these results highlight that the virtual screening approach successfully identified chemically diverse candidates, expanding the available chemical space for NLRP3 modulation and offering promising scaffolds for further experimental validation.

In drug design, ADMET refers to absorption, distribution, metabolism, excretion, and toxicity, and it is critical for determining drug efficacy, safety, and patient compliance [31]. In this study, ADMET predictions were performed for the top eight compounds with the highest shape similarity. Compound C8 exhibited high cell permeability and broad biodistribution, accompanied by greater molecular flexibility, which may facilitate target engagement but could also increase the risk of poor solubility, slow metabolism, and off-target toxicity. C1 and C6 exhibited higher water solubility, suggesting that injectable formulations should be prioritized in subsequent dosage form development. C1, C5, C6, and C7 are relatively more polar and display higher water solubility. C2 and C3 demonstrated improved intestinal permeability. Regarding BBB permeability, the predicted PlogBB values for compounds C1, C5, C6, and C7 were relatively low. In contrast, MCC950 has been reported to cross the blood–brain barrier and modulate neuroinflammation [32]. Notably, compounds C2 and C3 showed improved predicted intestinal absorption and moderate BBB permeability, suggesting their potential suitability for central nervous system targeting. Overall, C2, C3, and C4 showed the most balanced ADMET profiles, combining adequate solubility, permeability, and metabolic stability. In contrast, C1 may require further structural optimization to improve BBB permeability and balance hydrophilicity with lipophilicity. These insights provide a rational basis for prioritizing compounds for further preclinical development and formulation design.

To further explore the pharmacological potential of compounds C2, C3, and C4, we analyzed their chemical structures in the context of established NLRP3 inhibitors, such as MCC950 and Dapansutrile, which have been extensively characterized in terms of their pharmacophoric features. All three compounds contain multiple hydrogen bond donor and acceptor groups, including hydroxyl (–OH), amino (–NH2), and carboxyl (–COOH) functionalities. These groups can form hydrogen bonds with polar residues within the NLRP3 binding pocket, which is a key interaction feature also observed for MCC950 [33]. Aromatic or heteroaromatic rings present in C2, C3, and C4 provide opportunities for π–π stacking and hydrophobic interactions with aromatic or nonpolar residues in the binding site, similar to the interactions observed for the benzothiazole and pyridine moieties in MCC950 and Dapansutrile. Additionally, structural rigidity and planarity, conferred by ether linkages, fused rings, or rigid cyclic frameworks, may enhance binding stability by reducing conformational entropy upon ligand binding, a characteristic also shared by MCC950. Taken together, the combination of hydrogen bond donor/acceptor groups, aromatic systems for π–π stacking, and molecular rigidity forms pharmacophoric motifs in C2, C3, and C4 that are consistent with known NLRP3 inhibitors. These structural features likely contribute to favorable interactions within both polar and hydrophobic sub-pockets of the NLRP3 protein, supporting the potential of these compounds as modulators of NLRP3 inflammasome activity. This comparison provides a mechanistic rationale for the observed or predicted pharmacological activity and highlights the potential of these compounds as lead scaffolds for further optimization.

MD simulations were performed to predict the binding affinity and mode of interaction between the compounds and the protein based on theoretical calculations. Based on the shape similarity results, C2, C3, and C4 were selected for 200 ns MD simulations, as the physicochemical properties of C1 predicted by ADMET were suboptimal. RMSD, RMSF, Rg, SASA, and PSA analyses were performed to further investigate the stability of ligand binding within the protein. RMSD analysis indicated that all compounds reached equilibrium by the end of the MD simulations. The binding stability of C2, C3, and C4 was higher than that of the positive control XE3. RMSF analysis revealed that all four compounds exhibited higher fluctuations in the loop regions, indicating that these regions possess higher flexibility, whereas the helical regions remained relatively stable. The physicochemical properties of the compounds were further evaluated using Rg, SASA, and PSA. The Rg values of C2, C3, and C4 at 200 ns were approximately 6 Å, indicating that these molecules adopt a more compact conformation. C3 and C4 exhibited smaller SASA values, suggesting that they are more tightly solvated by the surrounding aqueous environment. C2 and XE3 showed lower PSA values, indicating enhanced potential for membrane permeability. Analysis of these physicochemical properties provides a theoretical foundation for the rational modification of small molecules. The binding free energies of the three screened compounds were superior to that of the positive control XE3. In particular, C3 exhibited the most favorable binding free energy of −48.81 ± 3.89 kcal/mol.

This study has certain limitations that should be acknowledged. Firstly, the NLRP3 NACHT domain used in this study is based on the experimentally resolved crystal structure (PDB IDs: 8WSM and 7ALV), which was employed to analyze the conformation of the NACHT domain and its interactions with small molecules. It should be noted that this structure only covers the NACHT region and does not include the full-length NLRP3 protein, which contains the PYD and LRR domains. Therefore, any insights regarding the overall conformation or inter-domain interactions of full-length NLRP3 still rely on homology modeling. Secondly, the current work relied entirely on in silico methodologies, including molecular docking, ADMET prediction, and molecular dynamics simulations. While these computational approaches provide valuable theoretical insights into the potential inhibitory mechanisms and stability of the identified compounds, they cannot fully substitute for experimental validation. Future work will therefore aim to experimentally validate the predicted NLRP3 inhibitory activity through in vitro and in vivo studies, such as measuring IL-1β secretion in macrophages and assessing caspase-1 enzymatic activity. These experimental efforts will be essential to confirm the biological relevance of our computational findings and to further advance the development of potent and selective NLRP3 inhibitors.

## 4. Materials and Methods

### 4.1. Database Integration

The small molecules in eight TCM databases, includes BATMAN-TCM [34], ETCM [35], HERB [36], HIT_2.0 [37], iTCM [38], SymMap v2.0 [39], TCMSID [40] and TCMSP [41], were integrated to a virtual library. The virtual library contained 146,730 TCM active ingredients, which were subsequently used as target library for screening.

### 4.2. Ligand Preparation and Protein Selection

A virtual library of 146,730 traditional Chinese medicine (TCM) ingredients was prepared using the LigPrep module in Maestro with the OPLS_2005 force field to generate optimized 3D structures. Possible ionization states were generated at pH 7.0 ± 2.0, with up to eight conformations per ligand.

The X-ray crystal structures of NLRP3 in complex with inhibitor (PDB IDs: 8WSM and 7ALV) were downloaded from the RCSB Protein Data Bank [42]. The structure with the highest resolution and intact binding site was selected for virtual screening. To validate the docking protocol, the co-crystallized ligand was redocked, and the RMSD between the docked and experimental poses was calculated, confirming the reliability of the docking procedure. Protein preparation included addition of missing residues, assignment of protonation states, hydrogen optimization, and energy minimization using the OPLS_2005 force field [43,44]. Docking grids were defined based on the co-crystallized ligand. To validate the docking protocol, the co-crystallized ligand was redocked and the RMSD between the redocked and experimental poses was calculated, confirming the reliability of the procedure. Glide docking was selected due to its validated performance in predicting binding modes and affinities of small molecules to protein targets, including NLRP3 [45].

### 4.3. Docking-Based Virtual Screening

Virtual library was imported to the module of Virtual Screening Workflow in Schrödinger 2015 for screening. The virtual library was pre-filtered according to Linpinski’s rule [46]. Then, QikProp module was applied to eliminate ligands with reactive functional groups. Finally, all ligands were docked into the binding site of NLRP3 protein in turn and evaluated step by step using the high throughput virtual screening (HTVS), SP and XP of Glide. In the screening process at each step, 20% of the best ligands were reserved for further analysis. All other options were left at their default values.

### 4.4. Shape-Based Screening

The Shape Screening module of Schrödinger 2015 focuses on identifying potential drug candidates by evaluating geometric similarities between molecules. Specifically, the structures obtained by VS were imported into the shape screening module for calculation. XE3 was used as the query structure in Shape Screening. Finally, Shape Sim was used to rank all the molecules. Shape-based screening was applied to complement docking by evaluating geometric similarity to known inhibitors, enhancing hit identification. Two-dimensional Tanimoto analysis provided additional assessment of chemical similarity to reported active compounds [47].

### 4.5. Similarity Calculations

The Tanimoto coefficient (Tc) was employed as the criterion for evaluating compounds using all two-dimensional (2D) similarity search approaches. Tc was calculated based on the ECFP4 fingerprint for XE3, the top eight compounds and twelve NLRP3 inhibitors (5J, 15Z, A1, CY-09, INF39, IZD-174, JC-121, MCC950, N14, OLT1177, RG6418, ZYIL-1) using the Canvas module of Schrödinger 2015 [48]. The module of Similarity Matrix From Finger was established the similarity matrix. And then, the module of Similarity/Distance Screen was used to calculate similarity within multiple ligands [49].

### 4.6. The Identification of Pharmacokinetics Properties

To predict the pharmacokinetic characteristics of the screened compounds, ADMET prediction was performed using SwissADME server and QikProp module of Schrödinger 2015 software [50]. The default mode is used for the primary descriptor. The following properties were evaluated: the octanol/water partition coefficient (QPlogPo/w), aqueous solubility (QPlogS), predicted IC_50_ value for blockage of HERG K+ channels (QPlogHERG), Caco-2 cell permeability (QPPCaco), and MDCK cell permeability (QPPMDCK). According to the definition of each parameter, the software gives a cut-off value to determine whether a certain parameter is suitable for the next screening.

### 4.7. Molecular Dynamics Simulation

Molecular dynamics simulations were employed to capture the dynamic behavior and conformational flexibility of protein-ligand complexes, providing a more realistic assessment of binding stability [51]. The dynamic behavior and stability of the protein-ligand complex and their binding were detected through MD simulations using the module of Desmond in Schrödinger 2015 [52]. Solvation was the first step of dynamics where ‘system builder’ was used for the same where Simple Point Charge (SPC) water model was employed in solvation [53]. An orthorhombic box was selected, and neutralization was performed by a fair number of sodium and chloride counter ions after calculations. The solvated system was then subjected to MD simulations for 200 ns with the standard default protocol by opting for periodic boundary conditions with NPT ensemble (Number of atoms, pressure, temperature). The temperature was adjusted to 310 K, pressure to 1.01325 atm [54]. Upon completion, a trajectory analysis was performed to obtain the RMSD, RMSF, Rg, SASA and PSA [55,56].

### 4.8. MMGBSA

MM-GBSA calculations were performed to estimate binding free energies and quantify interaction strengths, supporting the prioritization of compounds for further analysis [57]. The binding free energy calculation of the ligands targeting NLRP3 proteins was performed through Molecular Mechanics with Generalized Born and Surface Area (MMGBSA) solvation technique. After the trajectory of the last 20 ns of the MD simulation was extracted as 1000 frame, the ΔG binding energy of the protein-ligand complexes was calculated via Thermal MM/GBSA [58].

## 5. Conclusions

A total of 25 NLRP3 inhibitors were identified through a combination of DBVS and shape-based screening, all of which were derived from the active ingredients of traditional Chinese medicine. ADMET predictions of the top eight compounds exhibiting the highest shape similarity indicated that C2, C3, and C4 possessed the most favorable pharmacokinetic properties. MD simulation results demonstrated that C2, C3, and C4 were stably bound within the NLRP3 protein. Binding mode analysis revealed that ILE370, GLU369, and ASP662 were key residues mediating hydrogen bond interactions. Finally, binding free energy calculations indicated that C3, derived from the active component of American pokeweed, exhibited superior binding affinity compared with the positive control XE3. The elucidation of NLRP3 structure and its inhibitors provides novel insights into molecular-level interactions, offering a framework for the rational development of NLRP3 inhibitors with enhanced efficacy and specificity for chronic diseases.

## Figures and Tables

**Table 1:** The structural information of XE3 and top 8 with the best shape sim.

ID	PubChem ID	Name	Chemical Structure	Docking Scores (Kcal/mol)	Shape Sim	Source (English/Latin Name)	Database
XE3	NA	NA	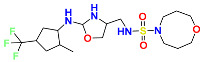	−8.057	1	NA	NA
C1	101010963	l-malicacid 2-o-gallate	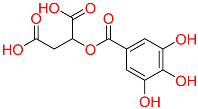	−10.522	0.328	*Phyllanthus emblica* L.	iTCM
C2	443775	Dioncophyllinol B	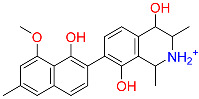	−10.802	0.305	*Akebia trifoliata*	HERB
C3	6444016	Isoamericanol A	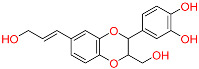	−10.119	0.280	*Phytolacca americana* L	HERB
C4	NA	NA	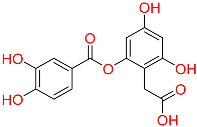	−10.137	0.251	NA	HERB
C5	135612764	Isobetanidin	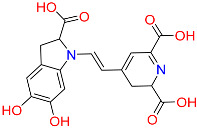	−10.129	0.247	*Portulaca grandiflora* Hook/*Cichorium intybus* L/*Portulaca oleracea* L/*Portulaca pilosa* L	HERB
C6	129716404	Monocaffeyltartaric acid	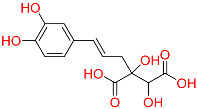	−11.277	0.229	*Cichorium intybus* L/*Monarda didyma* L/*Salvia coccinea* Buc’hoz ex Etl	HERB BATMAN-TCM iTCM SymMap V2.0
C7	135728	Gyrophoric acid	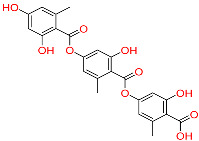	−10.669	0.226	*Gypsophila pacifica*	HERB
C8	71435823	Paludosicacid	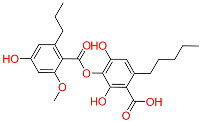	−10.161	0.217	*Silene jenisseensis*/*Parmelia* Lichen	HERB

**Table 2:** Estimated physicochemical and pharmacokinetic parameters by QikProp.

ID	PlogPo/w (−2.0–6.5)	PlogS (−6.5–0.5)	PlogHERG (<−5)	PPCaco (<25 Poor) (>500 Great)	PlogBB (−3.0–1.2)
XE3	1.948	−2.398	−6.186	61.217	0.107
C1	−0.581	−1.752	−0.75	0.236	−3.256
C2	3.007	−4.01	−6.167	270.285	−0.349
C3	1.48	−3.58	−5.528	114.108	−1.87
C4	0.17	−2.393	−3.152	2.603	−2.845
C5	1.113	−3.674	0.443	0.024	−3.905
C6	0.863	−2.055	−0.982	0.463	−3.089
C7	2.819	−4.59	−3.118	1.446	−3.127
C8	3.854	−5.275	−3.653	22.402	−2.559

**Table 3:** Binding free energy calculation of C2, C3, C4 through MMGBSA.

No.	ΔG Bind (kcal/mol)	ΔG Bind Coulomb	ΔG Bind H Bond	ΔG Bind Lipo	ΔG Bind vdW
XE3	−42.31 ± 5.31	−6.57 ± 5.45	−0.98 ± 0.66	−17.37 ± 1.55	−43.41 ± 3.72
C2	−34.17 ± 5.79	−81.64 ± 10.54	−1.20 ± 1.32	−19.64 ± 1.23	−41.06 ± 2.79
C3	−48.81 ± 3.89	−29.45 ± 5.13	−3.04 ± 0.56	−19.61 ± 1.76	−34.60 ± 2.77
C4	−33.50 ± 5.25	−3.64 ± 17.94	−2.21 ± 0.87	−13.45 ± 1.75	−29.05 ± 2.77

## Data Availability

The original contributions presented in this study are included in the article. Further inquiries can be directed to the corresponding author.
